# Unlocking the Black Box: The Molecular Dialogue Between ASFV and Its Tick Host

**DOI:** 10.3390/pathogens15010116

**Published:** 2026-01-21

**Authors:** Alina Rodríguez-Mallon, Thailin Lao González

**Affiliations:** Animal Biotechnology Department, Center for Genetic Engineering and Biotechnology, Avenue 31 Between 158 and 190, P.O. Box 6162, Havana 10600, Cuba; thailin.lao@gmail.com

**Keywords:** ASF, ASFV, *Ornithodoros* tick species, pigs, ASFV transmission, tick–host interface, ASFV infection

## Abstract

African Swine Fever is a lethal hemorrhagic disease caused by a DNA virus that affects domestic and wild pigs, causing serious economic losses in the swine industry. African Swine Fever virus (ASFV) is maintained in a sylvatic cycle that includes wildlife and *Ornithodoros* tick species. A huge investigation about ASFV structure and its infection process in pigs has been carried out in recent years, and although these studies have increased our knowledge about its pathogenesis, there are still many unclear aspects about which immune responses protect swine hosts against the disease caused by this virus. The mechanisms of ASFV infection in ticks are even less well understood. This infection is long term and persistent, with relatively high levels of virus replication in different tick tissues. According to specific infected tissues, the *Ornithodoros* tick species that are ASFV-competent vectors show transstadial, transovarial and/or venereal transmissions. This review is focused on the main process taking place at the virus–vector interface, summarizing the latest findings about the molecular and cellular aspects of ASFV infection in ticks, which could constitute the basis for developing novel strategies to interrupt the arthropod transmission cycle.

## 1. Introduction

African Swine Fever (ASF) is a highly contagious viral hemorrhagic disease that affects domestic and wild pigs, whose acute form can reach 100% mortality [1]. This disease is endemic to sub-Saharan Africa and was first described in Kenya in 1921 [2]. It is caused by a virus belonging to the *Asfarviridae* family (ASFV), which is classified into 24 genotypes and 8 serotypes, with different levels of virulence present only in Africa [3]. Transcontinental spreads of ASFV have included only genotypes I and II [1]. Currently, ASFV persists in Eastern European countries and Asia, with consequent devastating economic and productive losses to the swine industry [4,5,6,7,8]. To date, there are no available effective vaccines or treatments against ASFV due to the complex viral genome and its sophisticated ability to regulate the host immune response [9]. For this reason, timely and rapid diagnosis is essential to control the transmission of the disease; however, these measures have only limited success, and ASF has become a panzootic [10].

**Figure 1:**
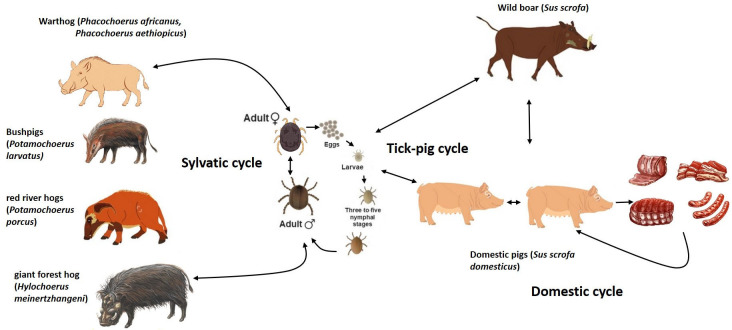
The life cycle of ASFV involving the tick and swine hosts.

The ASFV icosahedral particle has a multilayered structure with a 170–194 kb long linear dsDNA genome encoding more than 150 open reading frames [11,12,13]. It is the only known DNA virus transmitted by arthropods [14]. In Eastern and Southern Africa, the virus circulates in a sylvatic cycle, which includes wildlife reservoirs as warthogs (*Phacochoerus africanus*) that are infected by soft ticks of the genus *Ornithodoros* living in their warrens [15]. The common warthog can remain viremic for long time; however, it does not develop clinical disease because its viremia is usually low [16]. Therefore, ASFV transmission among these animals and domestic pigs is unlikely [17]. Other wild suids could be also involved in this sylvatic cycle, but they are considered to have a less significant role in ASFV dissemination to domestic pigs [14]. In this context, the tick–pig cycle is the most relevant for the ASFV outbreaks in domestic pigs. On the contrary, the contact of domestic pigs with infected wild boars is considered key within the ASFV domestic cycle in the Russian Federation, trans-Caucasian countries and Eurasia [17,18,19,20,21]. Wild boars (*Sus scrofa*) are native species in Europe, Asia and Northern Africa, and they are susceptible to ASFV infection, showing the same clinical symptoms and mortality rates as domestic pigs (Figure 1). In addition to all these dissemination ways, ASFV spreads over long distances as a result of viral survival in pork products stored even at negative temperatures [22].

Despite *Ornithodoros* sp. tick participation in the ASF epidemiology having been suspected since the first description of the disease [2], it was not discovered until 1960, during outbreaks in Spain in the 1960s, with *O. erraticus* as an actor in the ASFV’s sylvatic cycle in this region [23]. In Africa, it has been demonstrated that *O. moubata* complex soft ticks are able to maintain an ASFV infection for years and transmit it through sexual, transstadial and/or transovarial ways [24]. Nonetheless, vector competence for ASFV remains an investigation topic [25]. Not all Argasidae can transmit this virus, as only eight taxa from *Ornithodoros* ticks have demonstrated their ASFV biological vector abilities [26]. Results of these studies have demonstrated that the ability to maintain and transmit this virus is closely linked to the virus strain and the tick species [27]. To date, there is evidence that the ASFV is not able to replicate or be transmitted by hard ticks [28,29,30]. There are other studies where the capacity for ASFV transmission by different arthropods, including other blood-sucking insects, has been investigated and whose results have shown low probability for them to have an important role in long-term virus preservation and successful infection of susceptible pigs [31,32,33]. All these studies suggest a high specificity in the ASFV–tick interaction; however, viral and tick factors governing this interaction remain very limited even today. The scope of this review is, therefore, to synthesize current knowledge on the molecular mechanisms that underpin the complex interactions between ASFV and its tick vectors, highlighting gaps in knowledge and potential targets for intervention. To do this, PubMed (https://www.ncbi.nlm.nih.gov) searches with the terms “ASFV reviews” and “tick immunity”, as well as combinations of “ASFV” and “ticks”, “ASFV” and “pig” and “ASFV” and “tick saliva”, were performed on 15 August 2025 and on 17 October 2025 without date or language limitations. Duplicates were removed using EndNote 20 tools [34].

## 2. The Tick Vector: More than Just a Syringe

### 2.1. Biology of Relevant Ticks: Focus on Ornithodoros spp.

Despite the huge diversity of arthropod and arboviruses, the virus–vector combinations are very specific, suggesting precise molecular mechanisms involved in the viral infection and transmission by vectors, which are results from the long-term co-evolution of pathogens and vectors [35]. In the case of ticks, restrictions in vector competencies suggest main differences between tick families in their interactions with viruses [36]. To date, only eight *Ornithodoros* taxa in the family *Argasidae* have been described as biological vectors and reservoir hosts for ASFV [25]. Obviously, the specific biology of these species is relevant for the ASFV infection outcome.

Argasid (or soft) ticks lack a dorsal scutum in the adult and nymph stages; however the characteristics of their cuticle reduce water evaporation, allowing them to survive at high temperatures and under relatively dry conditions in tropical and subtropical zones and in arid areas of Central Asia and Africa, where ASFV is endemic and the highest genetic diversity of this virus is maintained in a sylvatic cycle that, up to now, includes only *Ornithodoros* tick species [15,37,38,39]. These species have an endophilous, nidicolous lifestyle living in a microhabitat in the burrows and caves of vertebrate animals that guarantee the required specific optimal conditions for their development [39]. Nymphs and adults of *Ornithodoros* species have short blood meals that can last from 15 to 60 min, which makes it unlikely to find them on their hosts. During this blood feeding, biological and mechanical ASFV transmission can happen. In addition, the high tegument distension and elimination of water excess and ions by coxal glands that occur during this feeding process lead to ASFV excretion in the tick vector’s coxal fluid, increasing tick-to-tick transmission [40].

Unlike ixodid ticks, soft ticks such as *Ornithodoros* species have between 2 and 8 nymph instars that require, just like larvae, at least one blood meal for molting. Their adults are long lived, with a maximum lifespan as great as 25 years for some species, and can feed up to 10 times when hosts are available [39]. Throughout their life cycle, these tick species are likely exposed to ASFV at multiple times depending on host availability and environmental conditions. In infected ticks, the virus can be isolated after many years post-infection [27]. In Madagascar, the presence of ASFV was detected in *O. porcinus* ticks in domestic pig premises unoccupied for at least 4 years [41]. Another study reported a longevity of *O. erraticus* ticks of 15–20 years and ASFV transmission after 5 years without a blood meal [42]. Additionally, female soft *Ornithodoros* ticks are iteroparous: they can lay eggs from two to five small clutches during their lifetime without previous copulation because they retain sperms within endospermatophores as an adaptation to host scarcity and/or climatic variability [43]. In addition, soft ticks, including *Ornithodoros* species, are very resistant to starvation, entering a quiescent phase or diapause for two to eight months during the winter or dry season until hosts become available [39]. All these characteristics of *Ornithodoros* spp. contribute to maintaining ASFV circulation in nature and explain why ASF eradication by pig vaccination is a challenging goal in areas where the sylvatic cycle is present.

Despite the vectoral competence of described *Ornithodoros* species to replicate ASFV, there are differences in viral titer and persistence according to the studied combination of soft tick species and virus strain [27]. Regardless of the fact that many of these results are incomparable due to bias of the different experimental designs used to obtain them, it has become clear that although a tick species can act as a reservoir of ASFV, infection with different viral genotypes could cause minimal cytopathological effects or even increase mortality and variations in the transmission efficiency [27,35,44]. For example, high mortality among *O. moubata* populations infected with some strains of genotype I (VIC T90/1 and Liv13/33) or genotype II (Georgia2007/1) has been observed. In addition, the Liv13/33 strain was vertically transmitted by this tick species but not the Georgia 2007/1 strain [27,36]. There is also evidence of ASFV clearance from infected *O. moubata porcinus* tick colonies that are fed with a virus-free blood. This fact could be explicated by the high mortality rates of infected ticks over the uninfected ones [45]. A study comparing fecundity in ASFV-infected and uninfected *O. moubata porcinus* and *O. erraticus* ticks showed that mortality rates were considerably higher among the infected ticks [45,46]. The high mortality of ASFV-infected ticks was also observed in *O. coriaceus* [31,47] and *O. moubata* (Murray, 1877) ticks [48]. On the other hand, *O. coriaceus* was able to transmit to pigs the Tengani/62 strain but not the Uganda/61 strain, while *O. porcinus* transmitted both strains [45]. In general, combinations between ASFV isolates not derived from ticks or *Ornithodoros* species not native from the place where the virus was isolated show increased tick mortality, suggesting that virus–tick adaptation could likely be necessary for successful ASFV persistence and transmission [18]. In this sense, molecular studies have suggested that the integration of ASFV genetic material in the genomes of soft ticks of the *O. moubata* complex occurred at least 1.47 million years ago and such long-term co-evolution could be responsible for the current high viral diversity present in Africa [16,49,50,51].

### 2.2. The Tick Midgut: The First Battlefield

Following the ingestion of ASFV-infected blood by ticks, their competence as vectors is defined by the successful replication and subsequent dissemination of the virus from the midgut to other tick tissues. Effective transmission will require the virus to infect specific target tissues, such as the salivary glands for horizontal transmission via saliva or the reproductive organs for vertical (transovarial) or sexual transmission [27,51]. Studies on *O. moubata* s.l. ticks have shown efficient ASFV replication in the midgut epithelium, circulating hemocytes, salivary glands and coxal glands [26]. This efficient ASFV multiplication points toward overcoming several barriers within the arthropod, such as the midgut peritrophic membrane, tick immunity and the resistance offered by the tick microbiota to pathogen invasions [52,53,54]. The gut represents the critical entry point that determines the success of the virus’ survival, replication and transmission. The midgut responsible for blood digestion, is additionally a key organ in the tick’s immune response against pathogens [35,36,40]. This tissue constitutes a microenvironment where viruses also come into direct contact with the resident microbial community, which can prevent or enhance the viral infection by direct interaction and/or modulation of the vector’s immune system components [55,56]. In this context, there is a study that has shown divergent midgut microbiome profiles (in composition, diversity and assembly) between *O. erraticus* and *O. moubata* s.l. tick species [57]. On the other hand, another study demonstrated ASFV sexual and transstadial transmission but no transovarial transmission in *O. erraticus* compared to *O. moubata* s.l. ticks, where all these transmissions have been demonstrated [46]. If demonstrated differences in microbiota between these tick species could underlie their distinct interactions with ASFV and drive the variations in their vector competence is an issue that remains to be investigated. An example of the impact of microbiota manipulation on the regulation of immune response and on ASFV infection vulnerability was demonstrated by the transplantation of African warthog (*P. africanus*) fecal microbiota to domestic pigs (*S. scrofa*), which conferred partial protection of these highly susceptible pigs against infection of attenuated ASFV strains [58]. Remarkably, most of the bacteria specifically found in transplanted pigs just before ASFV challenge have been associated with anti-inflammatory states and the production of AMPs in humans [59,60,61,62,63]. Therefore, microbiome transplantation from ASFV-resistant ticks to vector-competent *Ornithodoros* ticks could be a very interesting approach to evaluate the microbiota’s role in ASFV infection in ticks.

An experimental infection of *O. moubata porcinus* ticks with the Uganda or KWH/12 ASFV virus strain showed primary viral establishment, the highest titers and the longest persistence in the midgut [64]. Direct immunofluorescence studies of *O. turicata* ticks fed on an ASFV-infected animal revealed wide infection of the midgut epithelium after 2 or 3 days, while virus detection in the hemolymph and the salivary glands was possible only 20 days after feeding [31,40]. The results of another ultrastructural study using *O. porcinus* ticks infected with the Chiredzi/83/1 ASFV isolate showed that viral replication started in phagocytic digestive cells of the midgut epithelium, and 2–3 weeks later, virus infection was generalized to other tick tissues [40]. All these studies have demonstrated that efficient ASFV replication in the tick midgut is critical for virus infection generalization [65]. It means that once the virus has crossed midgut barriers, managing its replication in the cells of the midgut epithelium, it would be able disseminate to the salivary glands and reproductive organs, establishing a long-term, persistent and non-pathogenic infection in ticks.

## 3. Molecular Journey of ASFV in Ticks: A Stepwise Deconstruction

### 3.1. Viral Entry into Tick Cells

As mentioned before, in nature, ASFV circulates between individuals of different species, including domestic pigs, wild boars, warthogs and soft ticks; therefore, the virus has evolved to infect different cell types in each host. In swine hosts, ASFV has a restricted cellular tropism, targeting mainly macrophages and monocytes for replication [66], but also infects specific lineages of reticular cells in the spleen, lymph node, lung, kidney and liver [67]. In the same way, this virus is able to replicate and persist in different tick tissues, which demonstrates its adaptability for efficient spreading [26,65]. This selective pressure during host adaptation across evolution, geographical expansion and laboratory adaptation has determined the ASFV diversity for which deletions, insertions, inversions and duplications in its genome have been demonstrated [9].

The proteins encoded by the large double-stranded DNA genome of ASFV are classified into structural and nonstructural proteins, according to their roles in the viral particle. Structural proteins like pp220, pp62, p72, p54, p30 and CD2v are major components of viral particles and are crucial in virus assembly and in the interactions between the virus and its host cells [68,69]. They are codified by a conserved central region of the ASFV genome [70]. This central region is flanked by variable regions in both extremes that contain at least five multigene families (MGFs) coding nonstructural proteins that participate in cell tropism, viral replication, gene expression regulation, immune evasion and disease pathogenesis. These MGFs confer plasticity to the ASFV genome, allowing for strain diversification and the adaptations mentioned above [69]. In fact, ASFV avirulent strains frequently have significant deletions of MGF regions [9]. For example, Portugal NH/P68 and OURT88/3 attenuated ASFV strains show extensive deletions in the MGFs compared to the virulent Lisbon 60 (L60) strain [71]. In the same way, ASFV strains adapted to cell culture demonstrated characteristic MGF alterations [72,73,74]. These MGFs are also highly variable in ASFV strains circulating in wild boar and domestic pig populations and in different geographical regions [75,76,77]. Therefore, these genetic divergences allow for differentiation between geographically distinct ASFV strains and for tracking their spreading [78].

Deletions of MGF360 (*3HL*, *3IL* and *3LL*) regions impaired viral capacity to establish generalized infections in *Ornithodoros* vector ticks, preventing efficient viral transmission, which demonstrates their critical role in ASFV replication and persistence in tick tissues [26,67]. These genes, together with the MGF 530 genes, are also significantly involved in promoting survival and virulence in infected swine macrophages [79,80]. All this evidence points out that MGF360 genes as important factors for determining the ASFV host range. However, given the lack of homology of the products of these genes with other known proteins, it has been difficult to speculate on their roles in virus–cell interactions and how they modulate tick cell infection [67].

**Figure 2:**
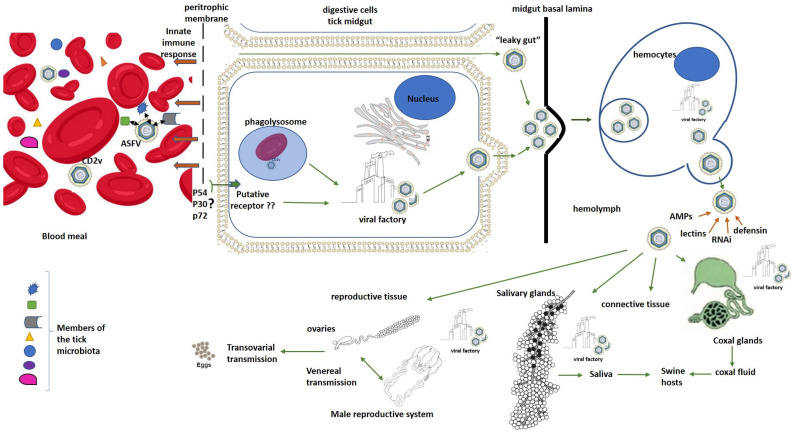
Schematic ASFV “molecular journey” inside ticks. Black arrows represent interactions between ASFV and microorganisms that are part of the tick microbiota inside the gut when infected blood is ingested by ticks. Brown arrows represent effects of the innate immune response mediators of ticks on ASFV. Green arrows represent hypothetical pathways for ASFV entry and dissemination inside ticks. The symbol ? means that the involvement of these viral structural proteins in a specific mechanism for ASFV entry to tick digestive cells remains to be demonstrated. In the same way, ?? means that the putative specific receptor for ASFV entry to tick digestive cells remains to be identified.

In ticks, erythrocyte digestion takes place inside microvilli of midgut digestive cells by phagolysosome formation [26,40]. In this regard, ASFV adhesion to erythrocytes could be crucial to enhance virus uptake and tick midgut cell infection [40]. The CD2v is a transmembrane and glycosylated protein of ASFV that has been described as essential in the viral hemadsorption (HAD) to red blood cells and as an enhancer of ASFV replication in *O. erraticus* ticks [81]. Therefore, adherence of viral particles to red blood cells could constitute a widespread mechanism enabling increased viral uptake and replication in arthropod vectors (Figure 2). Thus, the gene encoding this viral protein could be under strong positive selection in the sylvatic transmission cycle.

Nevertheless, many viral infections are initiated when the virus binds to a specific receptor in the host cells that triggers a complex cascade of intracellular signaling for virus internalization. The presence of such specific receptors on the luminal surface of the tick gut has not been demonstrated for ASFV entry and infection of tick digestive cells as an alternative mechanism for the processes of phagocytosis and pinocytosis described above. However, it could be supported by the fact that non-HAD mutant ASFV isolates can also enter and replicate in ticks [81]. The presence of this receptor could also be partly supported by the high specificity of ASFV replication in ticks only demonstrated in *Ornithodoros* species. The ASFV structural proteins, p54 and p30, have been demonstrated as key molecules in viral entry to host mammalian cells [82,83]. In the same way, the propeller-like top structure of p72 extended to outside of the virus has been likely implicated in the receptor-binding area on the pig cell surface [12]. Further studies will be necessary in order to elucidate if these proteins could be also involved in ASFV interaction with the putative receptors on tick cells. Other authors have also speculated a “leaky gut” phenomenon, in which the ASFV virus could pass directly into the hemocoel from the gut lumen through lesions or pores formed between the gut cells during the first phase of digestion without entering the gut cells [84,85]. However, this last theory contradicts some studies that have demonstrated that virus replication in the midgut epithelium is required for the generalization of viral infection in *Ornithodoros* ticks [27,65]. In this regard, other evidence reinforcing the essential role of the high viral replication in the midgut cells for ASFV dissemination and successful transmission by ticks is the speculation by some authors that it could be responsible for weakening the gut cell monolayer, making it susceptible to rupture, which is supported by the observations of gut rupture as a frequent cause of death in some ASFV-infected ticks [31,47,48]. A critical review of a study that suggested this “leaky gut” phenomenon for ASFV infection shows an experimental design in which ticks were artificially engorged on ASFV-infected blood in the presence of antibiotics and antifungals that could modify the integrity of the tick midgut, favoring the ASFV’s bypassing of midgut replication [27,84]. In any case, gaps in our understanding of ASFV infection and the dynamics of its dissemination in *Ornithodoros* ticks remain to be unraveled.

After midgut infection, the virus escapes from this organ to infect other tick organs using a not-well-described mechanism [36,86]. In soft ticks, the midgut basal lamina is a homogeneous and continuous layer that has neither been biochemically nor cytochemically characterized [26,40,65]. Possibly, ASFV spreading may involve virus movements across the midgut basal lamina into the hemocoel by the action of other molecules such as matrix metalloproteinases or through mechanical forces [26,40]. The delay in the generalization of ASFV infection in ticks may be explained by inefficiency in the translocation across this basal lamina and/or the need for high viral titers close to the basal lamina, which probably requires extensive virus replication and time [40].

Once in the hemocoel, the viral particles are picked up by the hemocytes, where they are efficiently replicated and propagated to other tick organs [26]. ASFV virions budding from the plasma membrane of infected hemocytes were observed [40]. ASFV presence was also demonstrated in hemocytes of infected *O. coriaceus* ticks by electron microscopy and immunofluorescence studies [87]. Secondary sites of virus replication in ticks included connective tissue, coxal glands, salivary glands and reproductive tissue [40]. To infect the salivary glands and be transmitted through saliva, the virus must cross a thin basal lamina that surrounds them [26,40]. ASFV particles have been detected at very high concentrations in the salivary secretions of *Ornithodoros* ticks, supporting this route as the main ASFV transmission path during tick feeding on hosts [26,40,64,88]. In addition, the fluids from the coxal organ can also contain a high concentration of the virus that can be delivered to the host during feeding [40,65,88]. In *O. porcinus porcinus* ticks infected with the ASFV Chiredzi/83/1 isolate, the virus replicated in the cells of both the filtration membrane and the collecting tubule within the coxal gland. Numerous virions were observed budding into the lumen of the filtration membrane [40].

Finally, ASFV can also infect reproductive organs in female and male ticks, which allows for transovarial and sexual transmission depending on the viral isolate and *Ornithodoros* species combination [26,46]. For example, *O. moubata porcinus* female ticks transmitted the virus to their offspring and also infected males and uninfected females [24,64]. However, *O. erraticus* female ticks infected with a strain isolated from Portugal transmitted the virus to naive pigs but did not display ASFV transovarial transmission or venereal transmission [24,46]. These results suggest the presence of a specific barrier in both the male and female reproductive tracts of this tick species for ASFV infection [46].

### 3.2. Viral Replication and Assembly in the Tick Environment

ASFV is a cytoplasmic virus whose DNA replication and morphogenesis take place in viral factories, localized near to the host cell nucleus and the microtubule organizing center [89]. In this localization, ASFV has no access to the transcription machinery within the nucleus of host cells, and therefore, its genome encodes proteins for its own transcription machinery [90]. Probably, this transcriptional independence of ASFV allows for its wide diversity of evolutionarily distant hosts. In these factories, the viral capsid assembles progressively until the formation of the mature viral particles [91,92,93]. It has been recognized that aggresomal pathways could be used by ASFV to concentrate viral proteins and facilitate replication and assembly in infected swine macrophages [89]. It is possible that this same pathway could be used in the replication and assembly of this virus in tick cells due to the fact that similar viral factories have been observed in midgut digestive cells and hemocytes of ASFV-infected *O. porcinus* ticks [26,65].

In tick cells, viral factories are observed in a uniform cytoplasmic region bordered by filaments in which there are virions at different maturation stages [26]. Frequently, abundant mitochondria are around these viral factories, found subjacent to the plasma membrane. This localization could contribute to the intracellular transportation of viral particles and their budding from the plasma membrane of host cells, giving rise to extracellular virions with an additional external membrane [12,26,40,65]. It has been demonstrated that both intracellular and extracellular ASFV virions are infectious, which indicates that the external membrane is not strictly necessary for infectivity [94]. Additionally, in tick cells, membranes from an unknown source associated closely with mature viral particles have been observed that probably could provide protection during long periods between feedings of the long-lived ticks [26].

On another hand, ASFV was also detected in salivary glands, being first observed in the connective tissue. Later, virus factories and mature virions were also present in the granular cells, with virions accumulating in secretory granules. In this context, virus replication increased 10,000-fold between 21- and 112-days post-infection [40]. Abundant budding and large numbers of mature virions with extensive viral factories are also observed in the filtration membrane and in the tubular portions of the coxal organ in infected ticks [26].

### 3.3. Step 3: Evasion of Tick Antiviral Defenses

When a pathogen comes into contact with ticks, the arthropod can offer resistance to infection, eradicating this pathogen, or tolerate it, with minimal impact on its health and fitness. This outcome will depend of many factors, such as the immune response required for combating the specific pathogen and its energetic cost compared to the pathology induced by such a pathogen along with co-evolution, the specific microbiota and environmental conditions [95]. In ticks, immunity is based only on an innate immune system, which comprises cellular and humoral responses. The cellular component involves hemocytes circulating in the hemolymph that fills the whole tick body and surrounds all cells. These hemocytes are homologous to the mammalian white blood cells in terms of their immune function, which includes phagocytosis, nodule formation and the encapsulation of invaders [96,97]. The humoral component includes humoral encapsulation, hemagglutination and antimicrobial peptides (AMPs). Similar to other eukaryotes, ticks encode signal transducers involved in signaling pathways that regulate the innate immune response, such as toll, immunodeficiency (IMD) and Janus kinase and also transcription activators of the JAK-STAT pathway [98].

Humoral encapsulation is elicited by the prophenoloxidase activating system, which has been described only in the soft tick *O. moubata* [99]. Tick lectins function in hemagglutination to enhance phagocytosis by tick hemocytes [53,97]. Studies about pathogen uptake by phagocytic hemocyte populations have suggested that in competent vectors, the pathogens utilize hemocytes for surviving and disseminating inside ticks [96]. Lysozymes, together with other AMPs, are involved in killing invaders in several tick species [97]. Additionally, cystatins, classic cysteine protease inhibitors, have recently been shown to also function in innate immune responses of ticks [97]. It has also been demonstrated that hemoglobin fragments have antimicrobial activity in the midgut of ticks. The combination of all these immune responses in each tick species against a specific pathogen determines the vectoral capacity for such pathogen of the tick species in question [96]. Understanding the complex interaction of cellular and humoral reactions that occurs when ASFV gets the first lines of tick defense in the integument and gut may help to answer questions about why some *Ornithodoros* tick species are vector competent for this virus and other ticks are not.

Agglutinins/lectins with molecular sizes from 30 to 85 kDa have been described from the hemolymph, hemocytes, gut and salivary glands of several soft tick species [53]. The lectin named Dorin M with high hemagglutinating activity was identified in the hemocytes and plasma of *O. moubata* ticks as a molecule for recognizing non-self in innate immunity and could also be involved in mechanisms of pathogen transmission; however, its role in the ASFV infection of this tick species is still unknown [100,101]. On the other hand, a lysozyme purified from the gut of *O. moubata* was significantly upregulated after blood meal [102,103]. However, it is unclear if this lysozyme only has a digestive function or if it is also involved in an immune response against ingested pathogens. Lysozymes were described to be active only in the gut of soft ticks but not in the gut of hard ticks [103]. However, they are upregulated in the hemolymph and hemocytes in hard ticks, which remains to be demonstrated in soft ticks [104]. Further studies are needed to elucidate the different action mechanisms of lysozymes in soft and hard ticks and how this may impact its different capabilities as ASFV vectors. Cystatins, for their part, have been found in hard ticks, but until now, there are no reports about them in soft ticks [97,105,106].

**Figure 3:**
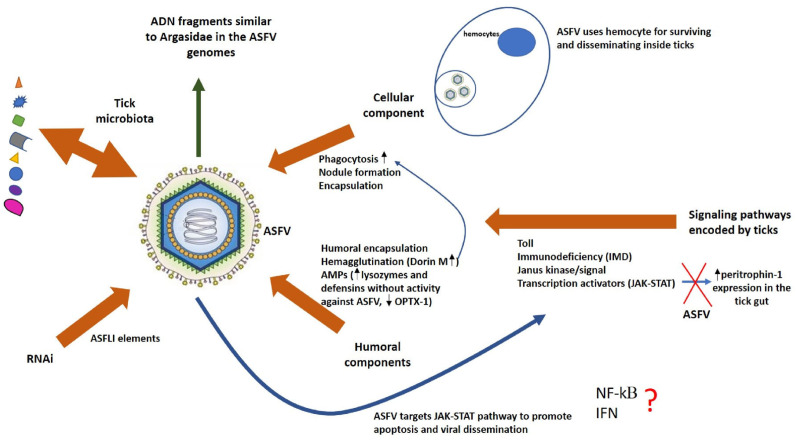
Schematic representation of ASFV interactions with the innate immune system of competent vector ticks. Brown arrows represent different effectors of the tick innate immune system that could influence the ASFV infection outcome. The wider blue arrow represents the putative effects of ASFV on the signaling pathways that regulate the tick innate immune responses. The thin blue arrows represent the effects of some mediators of the tick immune response over others. The green arrow represents putative mechanisms developed by ASFV to evade and suppress immune responses in their hosts. The symbol ? means that the ASFV interference with NF-kB role in the tick immune response and also if tick IFN response is associated with viral resistance in arthropods are issues that remain to be elucidated.

Finally, AMPs like defensins and their upregulation in the midgut and other tick tissues have been reported in *O. moubata* as part of a strong antibacterial response; however, their role in antiviral response remains to be more deeply studied [107,108]. A relatively recent work discovered a defensin-like peptide named OPTX-1 from *Ornithodoros papillipes* that inhibited the ASFV protease involved in structural protein processing, which is essential for its viral assembly and replication [109,110,111]. The analogs of OPTX-1 isolated from hard ticks were much more potent inhibitors of this protease, which could explain why ASFV is not able to replicate or be transmitted by hard ticks [28,29,30]. In another study on *O. turicata*, a putative vector for ASFV, four defensin genes were identified with different expression patterns between organs and physiological stages [112]. Some of them were homologous to defensins without antiviral activity against ASFV as described in *O. moubata*, which is one of the main vectors for ASFV transmission [109]. Additional studies are needed in order to validate if this is a real mechanism of ASFV vector competence of *Ornithodoros* sp. (Figure 3)

A proteomic study on *O. moubata* tick cells without or with ASFV infection, conducted to understand better the role of tick proteins in the interaction with ASFV, found 788 that were differentially expressed [113]. Among these, three of the top five upregulated proteins (ficolin, serine protease precursor and lysozyme precursor) have been reported to contribute to tick innate immunity against pathogens, and the implication of endocytosis in ASFV infection was highlighted by the notable downregulation of nine proteins of this pathway [100]. However, further assessments of this valuable information must be carried in order to validate the role of these proteins in ASFV replication and transmission in this arthropod vector.

RNA interference (RNAi) has been also described as the main antiviral mechanism in ticks [114]. It has been mainly studied in hard ticks, and it is speculated that it could interfere directly with the viral infection or regulate the production of AMPs [115]. However, there are reports in which the RNAi pathway maintains arboviral infection and vector competence for transmission [116]. Genomic studies on *O. moubata* and *O. porcinus* soft ticks identified sequences over 20 kb coming from the ASFV genome, which were named African Swine Fever virus-like integrated (ASFLI) elements. These germline integration events could be explained by ASFV infection of reproductive tissues of both tick species. However, the nucleotide sequence of the mapped reads in each *Ornithodoros* tick species had a different identity percentage with respect to the sequences of the different ASFV genotypes [50,117]. In addition, transcriptomic studies identified small RNAs corresponding to the ASFLI elements identified in each tick species [50]. All data about experimental infection of *Ornithodoros* tick species by specific ASFV genotypes and the ASFLI element-specific small RNAs taken together suggest that ASFLI elements could be involved in an RNAi-based mechanism of defense against ASFV infection in *Ornithodoros* ticks, which, in conjunction with other components of the tick immune system, could determine which ASFV strain can infect a specific *Ornithodoros* tick species [50].

In parallel, the viruses have also developed mechanisms to evade and suppress immune responses in their hosts. A large number of ADN fragments similar to hosts including both Argasidae and suids were found in the ASFV genomes [118,119]. Notably, these fragments were found more frequently in non-coding regions of the viral genome containing promoter sequences, aiding immune evasion and adaptation to the host [118,120]. All viral sequences that resemble those of the host suggest that ASFV may disrupt host cell processes by mimicking their proteins in order to support viral survival, facilitating replication and transmission [121]. Studies comparing genomes of ASFV strains of genotypes I and II identified an exclusive gene named X64R in some genotype I strains that is speculated to be involved in the specificity of the tick–virus combinations [27].

In pigs, it has been demonstrated that ASFV targets cGAS-STING, NF-κB, TGF-β, ubiquitination and apoptosis signaling pathways for promoting viral replication [68,122]. ASFV MGF-encoded proteins suppress interferon signaling, NF-κB and JAK/STAT pathways in susceptible swine hosts [9,122]. Due to the fact that ASFV also adapted to replicate in some soft *Ornithodoros* ticks and this infection can persist over years, it is also probable that proteins encoded by the viral genome could enhance virus replication and evade the arthropod defense system [81]. There are examples in which arbovirus activated the host JAK-STAT pathway to promote apoptosis, benefiting viral infection and dissemination in the arthropod vector, when, in general, apoptosis is considered to be an efficient defense against viruses in mammals [123,124,125]. This JAK/STAT pathway was demonstrated to be functional in ticks and also associated with AMP expression; however, its signaling mechanism is unclear because the receptor triggering this pathway has not been identified yet [53]. Interestingly, it was reported that *I. scapularis* ticks use a vertebrate host-derived cytokine to stimulate their own JAK/STAT immune pathway [126], and the STAT knockdown in this tick species decreased peritrophin-1 expression, affecting gut epithelium integrity [55]. In addition, a kappa B (kB)-binding region has been identified in the promotor region of certain insect AMP genes, and it is known that ASFV can regulate the inflammatory response in pigs by regulating NF-kB [53,68].

Several studies have suggested that the transmembrane and glycosylated CD2v protein of ASFV, which is required for hemadsorption, also plays an important role in immune escape and viral pathogenesis by regulating the JAK2-STAT3 pathway and inhibiting apoptosis to facilitate virus replication in pig cells [127,128]. Remarkably, there are significant morphological and functional similarities between pig macrophages and tick phagocytic digestive cells. As mentioned previously, ASFV MGF360 and MGF530 genes suppress interferon (IFN) response genes in primary swine macrophage cultures [129]. Although IFN response had not been associated with viral resistance in arthropods, these ASFV genes may be involved—directly or indirectly—in the survival of infected tick cells by blocking an interferon-like signaling pathway (Figure 3).

### 3.4. Step 4: Transmission to the Host

Salivary ASFV excretion during feeding has been described as the main route for transmission of this virus from ticks to swine hosts [64,88]. In salivary secretions of *Ornithodoros* ticks, abundant ASFV particles have been detected. In the same way, crystal arrays of condensate ASFV particles have been observed in granule-forming cells. This evidence supports the fact that ASFV could be delivered at very high concentrations into the host during feeding [26,40]. However, a study showed that ASFV transmission was successful when 30 infected ticks simultaneously bit a pig, whereas transmission failed when multiple tick challenges were carried out using 15 infected ticks each time, which demonstrated that viral loads and the quantitative presence of bioactive salivary gland molecules could play an essential role in the competence of *Ornithodoros* ticks as ASFV vectors and are additional factors to take into consideration for explaining transmission success [27].

It is known that tick saliva is a pharmacopoeia of biologically active molecules locally regulating host processes such as vasodilation, wound healing, platelet aggregation, blood coagulation, innate immune responses, the complement system and acquired immune responses for facilitating ticks with obtaining a blood meal from their vertebrate hosts [130,131]. Obviously, these interactions at the tick–host interface could also facilitate pathogen infection and proliferation in the hosts; however, there is little evidence about direct interactions between tick-borne viruses with the molecular constituents of tick saliva [36]. Results of a study in which pigs were co-inoculated with ASFV and salivary gland extracts from *O. porcinus* ticks showed enhanced macrophage recruitment at the inoculation site compared to pigs inoculated with the virus alone, which could promote viral infection, taking into account that macrophages are the main target cells in swine hosts [132]. Additional studies about the effect of saliva containing ASFV on the modulation of the immune cells and the expression levels for cytokines in different host tissues are necessary in order to improve our understanding about how the active components of saliva can facilitate viral infection, bearing in mind our previous knowledge about ASFV pathogenesis in pigs [68].

In general, the basic components of tick saliva are water, ions, non-peptide molecules, tick peptides, tick proteins, host proteins and exosomes. Studies about the salivary transcriptome and proteomics of the soft *Ornithodoros* tick species have found lipocalins, Kunitz, cystatin, basic tail, hebraein, defensin, TIL domain, metalloprotease, 5′-nucleotidase/apyrase and phospholipase families of proteins and have also identified protein families uniquely found in the Argasidae family, such as the adrenomedullin/CGRP peptides, 7DB, 7 kDa and the RGD-containing single-Kunitz proteins. Additionally, three other unique protein families common only to the *Ornithodoros* genus were discovered [133,134]. Moreover, it is known that *O. erraticus* tick bites cause greater inflammation and injuries in host tissues than those caused by ticks from the *O. moubata–O. porcinus* complex [135]. The role of all these proteins and specially that of those exclusive ones remains to be elucidated, in line with the ASFV vector ability of *Ornithodoros* tick species. Some studies have also suggested an important role of tick heat shock proteins (HSPs) and organic anion-transporting polypeptides in arthropod blood feeding and in interactions with the pathogens [136]. The knockdown of HSP70 expression in a tick cell line resulted in increased Langat virus replication [137]. It could be an interesting issue to address in order to know if reduced expression of HSPs is also associated with enhanced ASFV replication in tick cells.

In summary, all knowledge about the biology of tick feeding and molecular interactions at the tick–host interface is very important for understanding the mechanisms involved in successful pathogen transmission and will eventually allow us to develop vaccines to block it. Notably, despite the functions of multiple molecules in tick saliva being identified and their contributions to tick feeding success and host immune evasion being described, currently, around 80% of identified saliva proteins do not have a known function, and fewer than 5% of those proteins that have been functionally annotated have the putative function verified [131]. It is also probable that tick saliva contains a number of unknown proteins that have not yet been identified. This scenario is even more complex because there are studies that have shown that tick saliva is dynamic, and its protein expression profile changes according to host responses and when harboring viruses [138].

## 4. Comparative Mechanisms: ASFV in Ticks vs. Swine

Knowledge about the molecular mechanisms of ASFV infection in ticks remains scarce and poorly understood. Although the ASFV strategies to successfully infect vertebrate cells are better known, their extrapolation cannot be fully assumed in its persistent infection of tick cells, and further investigation is necessary to elucidate specific interactions of this virus with the molecular mechanisms of tick defenses. The Table 1 shows a comparison between the ASFV mechanisms in tick vs. pig hosts

## 5. Implications and Applications

Knowledge about the biology of *Ornithodoros* tick species that are competent for ASFV transmission and the molecular interactions at the tick–host interface during viral transmission is very important for developing disruptive strategies for its transmission cycle and blocking ASFV spread. Anti-tick vaccines targeting important components of tick saliva enhancing ASFV infection, crucial viral attachment molecules or transmission-blocking molecules could constitute interesting approaches to address these strategies and prevent this tick-borne disease [145].

In the same way, the combination of genomic, transcriptome/proteomic and metabolomic data can reveal genes and proteins from both the vector and the virus with important roles in persistent ASFV infection in ticks, which could also guide us in the design of attenuated ASFV strains as vaccine candidates to prevent lethal disease in pigs [146,147]. Functional genomic tools such as mutagenesis, RNAi and CRISPR could facilitate the identification and validation of these essential molecules in tick signaling pathways, as well as the use of modified ASFV strains to validate the specific factors identified as essential for its replication in tick vectors [148,149].

## 6. Conclusions and Future Perspectives

In summary, the road to fully understanding the underlying mechanisms involved in the vectoral capacity of *Ornithodoros* tick species for ASFV transmission is still long. Although in recent years a great amount of relevant knowledge about the tick–virus interface and the potential mechanisms implied in tick infection by ASFV and their transmission to vertebrate hosts has been gained, there is still limited understanding about the exact potential tick cell receptors for ASFV, the full repertoire of viral genes essential for tick infection, detailed tick immune responses to ASFV and mechanisms of transovarial transmission, among others. In this scenario, future research should be aimed at clarifying ASFV–tick interactions.

## Figures and Tables

**Table 1:** Mechanisms of ASFV in its tick vector versus its swine host.

Aspect	Tick Vector	Swine Host	References
Target cell types	Phagocytic digestive cells (initial infection)	Monocytes, macrophages and dendritic cells (primary targets)	[68]
	Systemic infection of hemocytes, salivary gland acinar cells, connective tissue and reproductive organs	Hepatocytes, endothelial cells and tubular renal epithelial cells	
Putative receptors	Largely unknown;	CD163;	[69,81]
	phagocytic pathway;	C-type lectins;	
	unknown specific receptors	Siglec-1 (CD169);	
		integrins	
Crucial viral genes	MGF 300/360;	EP402R (CD2v);	[68,139,140,141,142,143,144]
	EP402R (CD2v);	MGF360/505;	
	A179L;	A137R;	
	A224L	A238L; B646L (p72);	
		A224L (5EL) apoptosis; pC129R; pEP364R;	
		pE199L autophagy	
Crucial host genes	Genes for pH regulation in midgut;	Genes for type I IFN production and signaling;	[17,68]
	iron metabolism;	NLRP3 inflammasome;	
	innate immune pathways	C-type lectins	
Infection outcome	Persistent, chronic and non-lethal, with minimal cytopathology; efficient transmission by salivary secretions during feeding; transovarial and venereal transmission	Acute, severe, lytic and lethal disease in pigs; massive cytokine storm; hemorrhagic fever; intravascular coagulation; high mortality	[26]

## Data Availability

No new data were created or analyzed in this study.
